# A holistic approach toward development of plant-based meat alternatives through incorporation of novel microalgae-based ingredients

**DOI:** 10.3389/fnut.2023.1110613

**Published:** 2023-05-09

**Authors:** Allah Bakhsh, Juhee Park, Kei Anne Baritugo, Bosung Kim, Sung Sil Moon, Attaur Rahman, Sungkwon Park

**Affiliations:** ^1^Department of Food Science and Biotechnology, College of Life Science, Sejong University, Seoul, Republic of Korea; ^2^Healthy Food Technology, Sunjin Co., Ltd., Icheon, Republic of Korea; ^3^Department of Medicine and Therapeutics, Faculty of Medicine, The Chinese University of Hong Kong, Hong Kong, Hong Kong SAR, China

**Keywords:** plant-based meat, microalgae, spirulina, duckweed, yellow chlorella

## Abstract

This study explored the changes in the physiochemical, textural, sensory, and functional characteristics of plant-based meat (PBM) after incorporating novel plant-based ingredients including spirulina (SPI), duck Weed (DW), and yellow Chlorella (YC). In the chromaticity evaluation, the YC group (YCI YC2, and YC3%) displayed significant differences (*p* < 0.05) in lightness (L*) indices as compared to the control. Whereas, based on concertation gradient of SPI microalgae (SP0.5, SP0.7, and SP1%) incorporated into PBM patties demonstrated that SPI 1 had the lowest values (*p* < 0.05) in redness (a*) and yellowness (b*) followed by SPI 0.7 and SPI 0.5% concentration, respectively. The concentration gradient of the YC group indicated that YC3 was intended to be the highest crude fat value followed by YC2 and YCI. The ash content in PBM patties increased considerably (*p* < 0.05) as the concentration level of microalgae advanced in all treated groups. Based on the concentration level of YC incorporated microalgae into PBM patties indicated that YC 3 had the highest (*p* < 0.05) gumminess and chewiness while YC 1 had the lowest reported values in terms of gumminess and chewiness. Moreover, springiness and cohesiveness showed considerable differences between SPI and YC groups. In the sensory evaluation, SPI 1 showed the lowest value only in color and appearance (*p* < 0.05), conversely, the other sensory parameters were non-significant among all treatment groups (*p* > 0.05). The micronutrient in PBM presented an irregular pattern after incorporating various ingredients. However, levels were higher (*p* < 0.05) in the DW group (DW 0.5 DW 0.7, and DW% 1) than those in the other groups. Moreover, the SPI and YC groups showed detectable levels of diphenyl-1-picrylhydrazyl (DPPH) radical scavenging activity with, SP 1 showing the highest level of antioxidant activity. Acknowledging the limited research on PBM production, extraction technologies, and selecting various novel suitable ingredients in meat substitutes. Hence, to fill this knowledge gap an attempt has been made to incorporate various concentrations of microalgae including SPI, YC, and DW to enhance the quality and functionality of meat alternatives. To the best of our knowledge, this is the first report that describes the physiochemical, textural, sensory, and nutritional attributes of PBM incorporated with novel microalgae. Collectively these results indicate that the incorporation of SPI, DW, and YC may improve the quality of PBM without showing deleterious outcomes on the quality and functionality of the ultimate PBM products.

## Introduction

It is imminent that population growth and the subsequent increase in food demand will affect food markets worldwide (1). Therefore, the production of the PBM as a protein source is needed to reduce the widening gaps between traditional meat production and its higher consumer demand in the near future (2, 3). Meat plays a vital role in human nutrition, and red meat contains highly valued biological proteins with vitamins, iron, zinc, and other micronutrients (4–6). However, excessive intake of red meat increases the risk of type 2 diabetes cardiovascular complications, other metabolic disorders, and some forms of cancer (7–9). In contrast, approximately 50% of people, who consume vegetable-based diets, have a lower risk of metabolic diseases (9). Because of the potential health benefits of PBM, religious reasons, and environmental concerns with meat-based products, the demand for PBM is on the rise (10).

As the demand for sustainable-healthy food increases, PBM has become an innovative meat alternative because of its health-promoting functionality (11, 12). Important sources for PBM are soy, pea, and wheat protein, while mushrooms, rice, and wheat gluten have also been used in PBM to enhance meat-like characteristics (10, 13–15). To date, an extruded product, called Textured Vegetable Protein (TVP), is mostly used in PBM production because of its desirable physical characteristics as well as consumer satisfaction with the product (16–18). TVP is a by-product obtained from various plant proteins such as soy, pea, and wheat protein, through extrusion. It has been used to improve the textural profiles of meat and processed meat products (19).

Generally, vegetarian diets contain low levels of saturated fat and no *trans*-fat, but they lack micronutrients such as zinc, iron, and iodine (20). Notably, trace elements are known to regulate cell function to maintain homeostasis in the whole body. Regulatory activities of trace elements include heart protection, antioxidants, anti-inflammatory, and immune functions when the appropriate concentrations are applied (21). Subsequently, in recent years spirulina has attracted the attention of researchers because of its outstanding protein content, which is comparable to conventional meat (22). The use of spirulina in partial meat protein replacement can be beneficial to human health owing to its amino acid composition, absence of cholesterol, and high amounts of vitamins, minerals, essential fatty acids, polyphenols, and pigments (23). Likewise, dried duckweed contains 30% protein, 5% fat, and 7% starch, it has a strong anti-oxidative capacity because of the high level of lutein and zeaxanthin (24). Moreover, chlorella products also contain numerous nutrients and vitamins, including vitamin D and vitamin B12, which are absent in most plant-derived food sources. Dietary chlorella supplementation in mammals, including humans, has been reported to exhibit various pharmacological activities, including, antioxidant, antidiabetic, and antihypertensive effects (25).

Previously we have conducted a series of experiments on the production and development of PBMs with a detailed accent on texture, taste, and color characteristics (1, 4, 11, 15, 18). However, the available scientific literature is limited based on the current market projections and future demand for PBMs. Consequently, the current attempt has been made to incorporate the novel microalgae-based ingredient into PBM. Therefore, we hypothesized that the incorporation of appropriate binders, extenders, and other functional ingredients including spirulina, duckweed, and yellow chlorella may improve the overall palatability of PBM products. Hence, to the best of our knowledge, this is the first report that describes the physiochemical, textural, sensory, and nutritional attributes of PBM incorporated with novel ingredients.

## Materials and methods

### Raw material and extraction

Duckweed (DW) (echoherb, Chungcheongnam-do, Republic of Korea) protein was extracted using hot distilled water (90°C) for 1 h and diluted 20 times with distilled water (w/v) concentrated in a vacuum concentrator (EYELA, Buenos Aires, Argentina) and dried in a freeze dryer (LABOGENE, Allerød, Denmark) and evenly ground. Powdered DW was added to the PBM.

Spirulina (SPI) (INGREDIENTS BY NATURE, New Brunswick, NJ, USA), yellow chlorella (YC) (Daesang, Seoul, Republic of Korea), and DW were added to the PBM. YC replaced TVP amount by 1.0, 2.0, and 3.0%, and SPI and DW by 0.5, 0.7, and 1.0%, respectively. The new material mix was formulated using TVP, shiitake mushrooms, Isolate soy protein, tapioca starch, isolate wheat protein, smoked flavor, emulsion, garlic, emulsifiers, oils, color, seasoning, salt, and binders. The pre-mix ratios are listed in Table 1. The control group of the current experiment is red meat (round beef steak). The control samples were obtained from the local supermarket in Seoul Korea. The quality characteristics of novel PBM patties measured in the present experiment were compared with the beef patty control.

**TABLE 1:** Novel PBM patties formulae incorporated with microalgae proteins.

Ingredients %				Treatments (concentrations)					
	Spirulina			Duck weed			Yellow chlorella		
	0.5%	0.7%	1%	0.5%	0.7%	1%	1%	2%	3%
TVP	53.50	53.30	53.0	53.50	53.30	53.0	53.0	52.0	51.0
ISP	2.50	2.50	2.50	2.50	2.50	2.50	2.50	2.50	2.50
IWP	3.50	3.50	3.50	3.50	3.50	3.50	3.50	3.50	3.50
MC	1.0	1.00	1.00	1.0	1.00	1.00	1.0	1.00	1.00
Shiitake mushrooms	10.0	10.0	10.0	10.0	10.0	10.0	10.0	10.0	10.0
Tapioca starch	2.50	2.50	2.05	2.50	2.50	2.05	2.50	2.50	2.50
Smoked flavor	9.00	9.00	9.00	9.00	9.00	9.00	9.00	9.00	9.00
Emulsion (Lecithin+ oleogels)	12.0	12.0	12.0	12.0	12.0	12.0	12.0	12.0	12.0
Salt	0.20	0.20	0.20	0.20	0.20	0.20	0.20	0.20	0.20
Seasoning	0.90	0.90	0.90	0.90	0.90	0.90	0.90	0.90	0.90
Coconut oil	3.80	3.80	3.80	3.80	3.80	3.80	3.80	3.80	3.80
Spirulina	0.50	0.70	1.0	–	–	–	–	–	–
Duckweed	–	–	–	0.50	0.70	1.00	–	–	–
Yellow chlorella	–	–	–	–	–	–	1.00	2.00	3.00
Color	0.10	0.10	0.10	0.10	0.10	0.10	0.10	0.10	0.10
Garlic	0.50	0.50	0.50	0.50	0.50	0.50	0.50	0.50	0.50

Purified water 3 times of TVP. TVP, texture vegetable protein; ISP, isolate soy protein; IWP, isolate wheat protein; MC, methylcellulose.

### Sample preparation and processing

**FIGURE 1:**
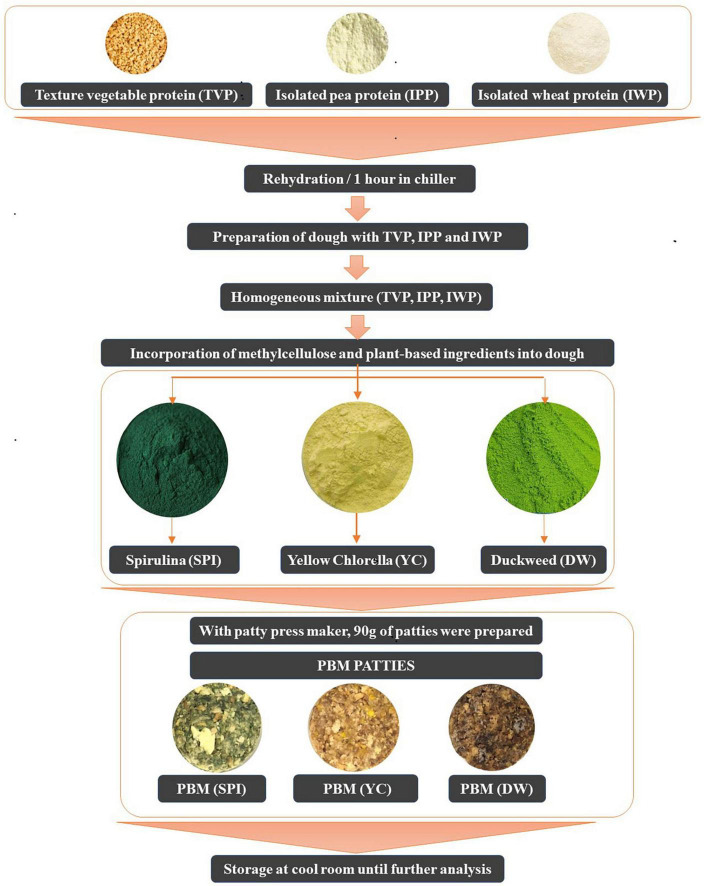
Flow diagram of PBM incorporated with novel microalgae ingredients.

**FIGURE 2:**
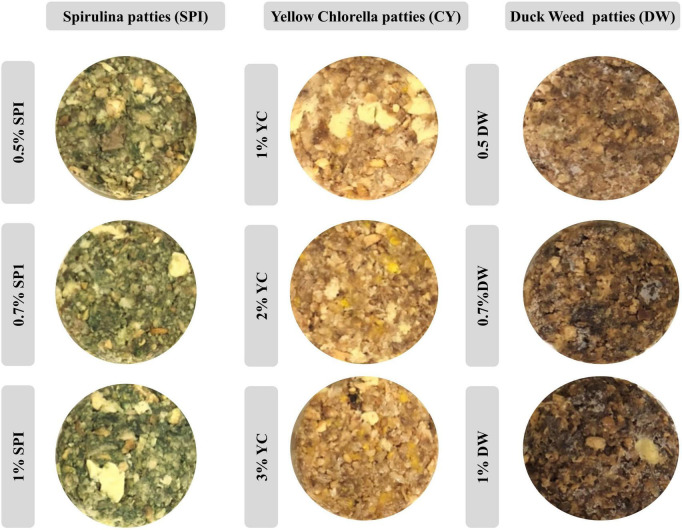
The visible appearance of PBM incorporated with novel microalgae ingredients.

Figure 1 shows the flow diagram of the PBM patties from processing to preparation. Briefly, a pre-specified volume of rehydrated TVP was added separately to the individual patty formulations. Consequently, for each patty formulation, novel ingredients including SPI, DW, and YC were independently incorporated into each mixture with TVP. All the ingredients listed in Table 1 were mixed using a Kitchen Aid mixer (Kitchen Art, Incheon, Republic of Korea) until a homogeneous mixture was obtained. Based on special considerations the pre-mixture was shaped into 90 g patties using a patty press maker (Hamburger Press Burger Patty Maker 304 Stainless, Incheon, Republic of Korea) with novel microalgae ingredients (Figure 2).

The present experiment consists of three major novel formulae (SPI, DW, and YC) and each novel formulation had three concentrations. Based on current specifications, six patties were prepared for each concentration with novel microalgae incorporated. Consequently, in total 54 patties were generated. In further classification, 27 patties were assigned to each raw and cooked batch, respectively. The patties were cooked at 180°C for 5 min on both sides with a non-sticking pan (Kitchen Art, Incheon, Republic of Korea). During the cooking process, the patties’ internal temperature was balanced using a probe thermometer until reached 75°C. The patties were allowed to cool for 30 min at room temperature. Subsequently, the physicochemical, textural, and sensory features were investigated.

### Visible appearance

The visible appearance of PBM patties incorporated with various concentrations of microalgae were photographed and analyzed using a digital camera (EOS 700D, Canon, Tokyo, Japan), and various structures were demonstrated (26).

### Color measurement

The surface color of the PBM was measured using a cross-section shaped on a 60 × 15 mm cell culture plate and measured with a colorimeter (KONICA MINOLTA, Chiyoda, Tokyo). The values were L*(brightness/lightness), a* (redness), b* (yellowness). The value of the standard white plate were 99.73 for L*, −2.3 for a*, and 6.23 for b*, respectively, and the chromaticity was measured from three random locations in each patty sample.

### Proximate composition

The proximate composition of the PBM was analyzed at Chungbuk National University (Chungcheongbuk-do, Republic of Korea). Approximately 100 g of PBM patty sample was used to determine the moisture content, crude fat, and ash content. The method for these analyses followed the Association of Official Analytical Chemists (AOAC) method (27).

### TPA

The textural attributes of PBM were determined using the following method. The patty sample of PBM dough was blended and molded, and 30 g of each on a 60 × 15 mm patty sample was added to the cell culture plate (SPL, Gyeonggi-do, Republic of Korea). The molded PBM was steamed for 10 min in a steamer preheated to 180°C and maintained at −20°C until use.

Furthermore, the PBM patty samples for texture profile analysis (TPA) were thawed at room temperature (20°C) and cooked evenly using an electric grill (Kitchen Art, Incheon, Republic of Korea). The test type was changed to TPA, Test Target Distance was 5.0 mm, and the Trigger Load was 9 N. This procedure was repeated in a triplicate way with the CT3 Texture Analyzer (Brookfield, Toronto, ON, Canada).

### Sensory evaluation

The sensory evaluation performance was followed by the sensory test guidance approved number SJU-HR-E-2019-010, Sejong University, Seoul, Korea. The sensory evaluation was conducted by nineteen trained students from the Department of Food Science and Biotechnology, at Sejong University. Accordingly, small pieces of different samples (2 cm × 2 cm × 2 cm) were prepared and random coding was assigned on a pre-positioned glass container (Pyrex, Charleroi, PA, USA).

The patty samples of PBM including SPI, DW, and YC were permitted at room temperature for 30 min and sequentially distributed among the panelists. To accomplish standardization, the special cabins assigned for sensory evaluation were provided with fluorescent light and sensory attributes were judged in a triplicate way by each panelist. The nine-point scale (1 = extremely weak or dislike, 9 = extremely strong or like) was used to evaluate, and Color, Odor, Flavor, Appearance, Oiliness, Juiciness, Chewiness, Springiness, and Overall acceptance (28).

### Micronutrients analysis

Micronutrient analysis was requested from Chungnam National University’s Agricultural Science Research Institute, and the analysis was conducted according to the following method. The mineral contents of Na, K, Zn, Mn, Fe, and Mg were analyzed by preparing a sample using the dry painting method. Samples of 2–5 g of PBM were placed in a self-made crucible, preliminarily preheated on a hot plate, incubated at 600°C for 2 h, and then cooled. Next, quantitative filter No. 6 (Advantec Co., Tokyo, Japan) was filtered using hot water, filtered to 100 ml, and analyzed using ICP-OES (iCAP 7400, THERMO). All reagents and distilled water were used for the mineral analysis.

For analysis As and Cd of samples, put a certain amount of sample into the microwave (QWAVE 2000, Questron technologies corp.), decompose with 70% nitric acid, give a quantification to 100 ml, and use ICP-OES (iCAP 7400, THERMO) analysis was carried out. All reagents and distilled water were used for purpose of mineral analysis. Mercury (Hg) was analyzed using a Mercury analyzer (DMA-80 Milestone) and calculated using a calibration curve obtained as a standard solution.

### BCA

Bicinchoninic acid protein assay (BCA) analysis was performed to confirm the protein concentration of PBM containing novel microalgae ingredients (29). PBM with SPI, YC, and DW added in different content was used for the analysis. Each sample was lyophilized and then crushed. 0.1 g of the sample was taken with 1 ml of RIPA buffer (Sigma-Aldrich, St. Louis, MO, USA) and vortexed for 10 s. The tube was placed on ice and stored in the refrigerator for 20 min. Samples were run for 10 min at 4°C, at 12,000 rpm in a centrifuge. Store at −70°C until used.

Samples already diluted to 10^1^ were diluted with sterile distilled water (D.W) until 10^3^. Using the Pierce BCA Protein Assay Kit (Thermo Fisher Scientific, Waltham, MA, USA), 25 μl samples and a 200 μl working agent (50 parts of bicinchoninic acid and 1 part of CuSO_4_ solution) were distributed to the 96-well plate. At this time, the standard was Bovine Serum Albumin (BSA), and a concentration of 0–250 μg/ml was used as a standard. The plate was mixed for 30 s and left for 30 min in a 37°C incubator. Photographed at 562 nm in a Microplate reader.

### DPPH radical scavenging activity

diphenyl-1-picrylhydrazyl (DPPH) radical scavenging activity was performed by modifying the methods of Thaipong et al. (30) and Moon et al. (31). Each lyophilized sample was finely ground and 0.1 g was taken and mixed with 1 ml 50% ethanol. The supernatant was recovered by centrifugal separation at 15,000 rpm for 10 min. The extraction process were conducted at room temperature for 12 h and the resultant extract were stored at −20°C until further analysis.

A 0.2 mM DPPH solution was prepared by taking 7.8 mg of 2.2-Diphenyl-picrylhydrazyl (Sigma-Aldrich, St. Louis, MO, USA) and 100 ml of methanol. The sample groups already diluted with methanol and 10^1 were diluted to 10^4 and distributed 20 μl of the sample in 96-well plates and 200 μl of the DPPH solution. Absorbance was measured at 517 nm in a microplate reader. Currently, the reference material was L-ascorbic acid (Sigma-Aldrich, St. Louis, MO, USA) and the control was 50% ethanol. Standard concentrations were 0–250 μg/ml. DPPH free radical scavenging ability was calculated by the following equation.


$$\mathrm{Percentage}\mathrm{of}\mathrm{inhibition}\left(\%\right)=\frac{\mathrm{Absorbance}\,\mathrm{of}\,\mathrm{extract}}{\mathrm{Absorbance}\,\mathrm{of}\,\mathrm{DPPH}\,\mathrm{Solution}}\times 100$$

### Statistical analysis

Before analysis, Shapiro–Wilk’s and Levene’s tests were conducted to assess the normality and homoscedasticity assumptions. The results of PBM based on SPI, YW, and DW content are represented as the mean plus/minus standard deviation (SD). Analysis of variance (ANOVA) was conducted to verify significant differences among the groups at the significance level of *p* < 0.05 with R studio (Ver. 4.0.2, NA, USA). All the experiments were conducted in a triplicate way. For multiple mean comparisons, Tukey’s test was run at the level of 5%.

## Results and discussion

### Visible appearance

The visible appearance and color features of PBM patties incorporated with various types and concentrations of microalgae are shown in Figure 2. Consequently, patties incorporated with higher concentrations of SP (SPI 1, SPI 0.7, and SPI 0.5%) have displayed a more intense green color with a declining trend as concentration reduces. Likewise, concentration-oriented yellowish features were recorded for YC (YCI, YC2, and YC3%) with drifting toward light yellow coloration. Similarly, DW (DW1, DW0.7, and DW 0.5%) showed a brownish tinge toward light brown coloration as the concentration of novel incorporated ingredients increased.

Inconsistent with the current result previously (32) reported similar results in microalgae biomass as an alternative ingredient in cookies with intense greenish coloration in the final product. Whearse, beef patties incorporated with pulses and microalgal protein (chlorella and spirulina) showed a brownish opted visible appearance (22). Likewise, the visible appearance in broccoli soup incorporated with various concentrations of microalgae including SPI (0.5–2.0%) YC (0.5–2.0%), and Tetraselmis (0.5–2.0%) were reported (33). These authors further explained that the color differences between the control and the microalgae-containing soups were visible to the human eye, especially for soups prepared using SPI. Moreover, our previous studies indicated that cooking methods and various ingredients particularly binding agents and natural colorants play important role in the visible appearance of PBM patties (1, 15, 18). The profound variations in the visible appearance of food products could be due to higher pigment degradation with the baking process or with a pigment saturation effect, above certain microalgal protein concentrations.

### Color coordinates

The color coordinates of the PBM are enumerated in Table 2. The lightness (L*) values of PBM was significantly lower in the DW 1.0 and the YC group tended to have higher indices. A declining trend was observed in all PBM compared to the control. Likewise, various concentrations of SPI and DW added in PBM suggesting a lighter and greener coloration. However, yellowness (b*) was significantly different between the control and DW 0.7 (*p* < 0.05). YC 3 had the highest b* values and the lowest values were reported for DWI (*p* < 0.05). The vast dissimilarities among color coordinates in PBM might be due to the different colors (green, blue-green, and yellow pigments) of the protein extracts from the microalgae species.

**TABLE 2:** Color indices of PBM incorporated with microalgae ingredients.

Parameters	Treatment									
	Control^1^	SPI 0.5^2^	SPI 0.7^3^	SPI 1^4^	DW 0.5^5^	DW 0.7^6^	DW 1^7^	YC 1^8^	YC 2^9^	YC 3^10^
L*	−61.53 ± 1.37^bc^	−60.37 ± 2.25^b^	−63.1 ± 2.69^bc^	−64.97 ± 0.3^bcd^	−61.13 ± 5.42^bc^	−66.2 ± 4.11^cd^	−61.13 ± 3.14^d^	−55.37 ± 0.8^a^	−55.13 ± 1.82^a^	−53.73 ± 1.38^a^
a*	11.1 ± 0.9^a^	6.5 ± 1.15^c^	5.17 ± 0.55^d^	4.13 ± 0.23^d^	9.7 ± 1.04^ab^	9.57 ± 0.57^b^	9.23 ± 0.23^b^	9.77 ± 1.05^ab^	10.47 ± 0.57^ab^	10.47 ± 0.64^ab^
b*	12.77 ± 0.15^abc^	11.83 ± 2.42^abcd^	11.23 ± 0.9^abcd^	9.63 ± 0.95^cd^	12.63 ± 2.46^abc^	12.63 ± 1.45^d^	10.03 ± 3.38^bcd^	12.43 ± 2.31^abc^	13.5 ± 1.25^ab^	14.77 ± 0.85^a^

The results shown as mean ± standard deviation (n = 3). ^a,b,c,d^Means with the different letter are significantly different (p < 0.05). ^1^Control: PBM without treatment, ^2^SPI 0.5: 0.5% of Spirulina in PBM, ^3^SPI 0.7: 0.7% of Spirulina in PBM, ^4^SPI 1: 1.0% of Spirulina in PBM, ^5^DW 0.5: 0.5% of Duck Weed in PBM, ^6^DW 0.7: 0.7% of Duck Weed in PBM, ^7^DW 1: 1.0% of Duck Weed in PBM, ^8^YC 1: 1.0% of Yellow Chlorella in PBM, ^9^YC 2: 2.0% of Yellow Chlorella in PBM, ^10^YC 3: 3.0% of Yellow Chlorella in PBM.

One of the most prominent characteristics of microalgae combined in PBM products are color features. Apart from chlorophyll, which is the major photosynthetic pigment, microalgae also contain phycobiliproteins and a wide variety of carotenoids. Chlorophyll, the primary photosynthetic pigment in all algal biomass, can be used in food products and pharmaceuticals because of its anti-inflammatory, and antioxidant properties (34). For instance, Phycobiliproteins are green-pigmented, a fluorescent antioxidative agent that existed in the form of phycocyanin in SPI (35). Similarly, lutein and β-carotene, zeaxanthin, neoxanthin, violaxanthin, and chlorophyll are present as natural pigments present in YC (36, 37). Whearse, DW are abundant in Chlorophyll, Carotenoid, and α-Tocopherol (38). Previous literature indicated that higher levels of algal protein concentrations have considerable effects on the color characteristics of the final product (39–41). Microalgae like YC have an abundance of carotenoid pigments resulting in yellow coloration in the final product (42, 43). In supporting literature previously (44) combined microalgae (30%) and soybean protein tissue to improve the nutritional value, appearance, and color properties. Similarly, extrusion and extraction process algal proteins such as YC and SPI, have also been tested in the manufacturing of PBM alternatives with enhanced color characteristics (45–47).

Moreover, the differences in color indices were probably caused by the browning effect, which can be promoted by Maillard reactions (2, 22, 48). The Maillard reaction in these two manufacturing processes can lead to an undesired color and flavor formation in plant-based foods. The main coloration of plant-based proteins is the formation of melanoidin’s on the furan backbone (49–51). This protein coloration is an irreversible condensation reaction between the carbonyl groups of melanoidins and the amino groups of lysine and arginine. Therefore, phenolic compounds with hydrophobic interactions with plant-based proteins are also considered the cause of coloring (52). The redness (a*) of PBM in our current study was highest in the SPI group and no difference was found among the other treatment groups. Furthermore, the lower color and saturation indices in the treated group can be attributed to chlorophyll pigments as SPI and DW are chlorophyll-rich (53–55). However, the degree of redness of the SPI and DW depends on the types of extraction or processing. Brightness and yellowness were higher in the YC group because the color of YC itself was brighter than SPI and DW. The current results of color indices are consistent with the color coordinates of plant-based meat analog patties in relation to beef and pork patties and plant-based meat analog patties incorporated with red yeast and lactoferrin, respectively (1, 15).

### Proximate composition

The proximate composition of the PBM incorporated with the novel ingredients is presented in Table 3. The overall moisture content did not differ significantly (*p* > 0.05) between the treated and control samples. However, a trend of decrement was observed in moisture content with the addition of a higher concentration of microalgae with the negligible statistical difference among treated and control groups. Similarly, there was no significant difference in crude fat content between the treated and control group except for YC3 treated groups which exhibited significantly higher crude fat content than the control. Additionally, the ash content values are expressively higher in treated groups SPI 1 (17.93%), DW 1 (18.28%), and YC 3 (16.64%) than in the control (9.76%), respectively.

**TABLE 3:** Proximate composition of PMB incorporated with microalgae ingredients.

Parameters	Treatment									
	Control^1^	SPI 0.5^2^	SPI 0.7^3^	SPI 1^4^	DW 0.5^5^	DW 0.7^6^	DW 1^7^	YC 1^8^	YC 2^9^	YC 3^10^
Moisture content (%)	11.76 ± 1.59^a^	12.33 ± 1.44^a^	9.63 ± 2.28^a^	10.0 ± 1.49^a^	10.23 ± 0.99^a^	8.9 ± 0.58^a^	4.17 ± 3.54^b^	11.18 ± 3.72^a^	10.81 ± 0.89^a^	10.81 ± 0.86^a^
Crude fat (%)	5.37 ± 1.65^b^	6.76 ± 2.52^ab^	7.42 ± 0.45^ab^	8.1 ± 0.56^ab^	7.22 ± 1.65^ab^	9.32 ± 5.05^ab^	8.86 ± 0.62^ab^	6.55 ± 0.51^ab^	8.55 ± 1.02^ab^	9.73 ± 3.42^a^
Ash (%)	9.76 ± 1.84b^c^	8.41 ± 2.17^c^	9.56 ± 0.06^bc^	17.93 ± 1.65^a^	8.25 ± 1.67^c^	14.51 ± 3.97^abc^	18.28 ± 3.32^a^	14.05 ± 6.21^abc^	14.97 ± 4.9^abc^	16.64 ± 2.58^ab^

The results shown as mean ± standard deviation (n = 3). ^a,b,c^Means with the different letter are significantly different (p < 0.05). ^1^Control: PBM without treatment, ^2^SPI 0.5: 0.5% of Spirulina in PBM, ^3^SPI 0.7: 0.7% of Spirulina in PBM, ^4^SPI 1: 1.0% of Spirulina in PBM, ^5^DW 0.5: 0.5% of Duck Weed in PBM, ^6^DW 0.7: 0.7% of Duck Weed in PBM, ^7^DW 1: 1.0% of Duck Weed in PBM, ^8^YC 1: 1.0% of Yellow Chlorella in PBM, ^9^YC 2: 2.0% of Yellow Chlorella in PBM, ^10^YC 3: 3.0% of Yellow Chlorella in PBM.

Previously, (56) and (18) reported that PBM analog products have higher moisture content in treated patties than in controls. Additionally, the authors described that the rehydration process, led the TVP to captivate extra water as methylcellulose gelatin, and starch tends to adsorb excess water compared to the meat and muscle system. However, the available reports supports our results in noodles and tofu with a slight decline in moisture content and an increased level of spirulina addition (53, 57). Based on current observations regarding the incorporation of microalgae species in PBM, (58) reported 1.1% crude fat content in SPI and (59) testified 5.02% crude fat in DW. The large variations in the PBM products and control could be due to the non-homogeneous emulsion fat gel in the PBM. Similarly, SPI, DW, and YC have high mineral contents which is consistent with our results (58–60).

### TPA

The TPA results for PBM are described in Table 4. Chewiness and Gumminess were similar between the control and SPI 1 groups, whereas other indices were significantly different (*p* < 0.05). Hardness and fracturability were similar among experimental groups, while there was a significant difference in DW 0.5 and YC 3 (*p* < 0.05). Similarly, springiness and cohesiveness showed significant differences among the treatment (SPI, and YC groups) (*p* < 0.05). The adhesiveness of DW 0.5 to 0.7 and YC 2 was significantly different from that of the control.

**TABLE 4:** Texture profile analysis of PBM incorporated with microalgae ingredients.

Treatment	Texture profile							
	Chewiness (N × mm)	Hardness (N)	Fracturability (g)	Gumminess (N)	Springiness (mm)	Adhesiveness (N⋅s)	Cohesiveness (%)	Resilience (%)
Control^1^	66.17 ± 4.24^b^	49.60 ± 1.96^bc^	49.60 ± 1.96^bc^	23.60 ± 1.22^b^	2.80 ± 0.08^a^	0.20 ± 0.00^a^	0.47 ± 0.01^a^	0.12 ± 0.01^ab^
SP I 0.5^2^	41.83 ± 3.45^c^	44.16 ± 1.96^cd^	44.16 ± 1.96_*cd*_	17.32 ± 0.68^cd^	2.41 ± 0.12^c^	0.07 ± 0.06^ab^	0.39 ± 0.00^de^	0.11 ± 0.01^ab^
SPI 0.7^3^	42.07 ± 7.07^c^	41.57 ± 2.90^cd^	41.57 ± 2.90^cd^	16.84 ± 1.80^cd^	2.49 ± 0.15^bc^	0.07 ± 0.06^ab^	0.4 ± 0.02^d^	0.11 ± 0.01^ab^
SPI 1^4^	66.67 ± 7.57^b^	56.64 ± 4.74^ab^	56.64 ± 4.74^ab^	23.50 ± 2.30^b^	2.83 ± 0.08^a^	0.07 ± 0.06^ab^	0.42 ± 0.01^bcd^	0.11 ± 0.01^ab^
DW 0.5^5^	42.57 ± 3.88c	38.74 ± 4.73^d^	38.74 ± 4.73^d^	17.15 ± 1.24^cd^	2.73 ± 0.08^ab^	0.03 ± 0.06^b^	0.45 ± 0.01^abc^	0.11 ± 0.01^ab^
DW 0.7^6^	45.93 ± 3.84c	42.04 ± 6.03^cd^	42.04 ± 6.03^cd^	17.61 ± 1.40^cd^	2.61 ± 0.08^abc^	0.03 ± 0.06^b^	0.46 ± 0.02^a^	0.12 ± 0.01^a^
DW 1^7^	50.53 ± 7.87c	42.33 ± 4.17^cd^	42.33 ± 4.17^cd^	18.57 ± 2.36^c^	2.39 ± 0.31^c^	0.17 ± 0.15^ab^	0.41 ± 0.04^cd^	0.1 ± 0.02^bc^
YC 1^8^	27.27 ± 3.99_*d*_	42.60 ± 1.42_*cd*_	42.60 ± 1.42^cd^	14.49 ± 1.33^d^	1.87 ± 0.11^d^	0.10 ± 0.10^ab^	0.34 ± 0.03^f^	0.08 ± 0.01^c^
YC 2^9^	38.50 ± 12.8^c^	48.66 ± 6.87^c^	48.66 ± 6.87^c^	17.94 ± 3.67^cd^	2.11 ± 0.30^d^	0.03 ± 0.06^b^	0.36 ± 0.03^ef^	0.1 ± 0.02^bc^
YC 3^10^	78.13 ± 2.32^a^	62.61 ± 4.74^a^	62.61 ± 4.74^a^	28.14 ± 0.94^a^	2.78 ± 0.06^ab^	0.07 ± 0.12^ab^	0.45 ± 0.02^ab^	0.11 ± 0.02^a^

The results shown as mean ± standard deviation (n = 3). ^a,b,c,d,e,f^Means with the different letter are significantly different (p < 0.05). ^1^Control: PBM without treatment, ^2^SPI 0.5: 0.5% of Spirulina in PBM, ^3^SPI 0.7: 0.7% of Spirulina in PBM, ^4^SPI 1: 1.0% of Spirulina in PBM, ^5^DW 0.5: 0.5% of Duck Weed in PBM, ^6^DW 0.7: 0.7% of Duck Weed in PBM, ^7^DW 1: 1.0% of Duck Weed in PBM, ^8^YC 1: 1.0% of Yellow Chlorella in PBM, ^9^YC 2: 2.0% of Yellow Chlorella in PBM, ^10^YC 3: 3.0% of Yellow Chlorella in PBM.

It is essential to regulate and determine the textural attributes of PBM, as these features determine the eating quality of the ultimate products. The incorporation of novel microalgae-based ingredients tremendously affected the texture profile of PBM. The integration of novel ingredients enhanced the chewy sensation in all groups as the content of the treatment increased and SPI 1 and YC 3 have a stronger chewing sensation than the control. This is because protein-rich ingredients influenced the strengthening of the gluten network (57). The current results are also supported by Fradique et al. (61) who described that the addition of microalgae pasta had higher firmness than other treated samples. Additionally, (57) reported an increase in hardness, springiness, cohesiveness, gumminess, and chewiness values in raw noodles mixed with Spirulina. The detrimental factor related to higher values of texture and chewing feeling can be caused by a decrease in the moisture level, which may reduce the feeling of sloppiness and resolve the heterogeneity in the mouth (53, 57). However, in contrast to our findings (22) reported insignificant textural attributes in beef patties replaced with pules and microalgal proteins.

### Sensory evaluation

**FIGURE 3:**
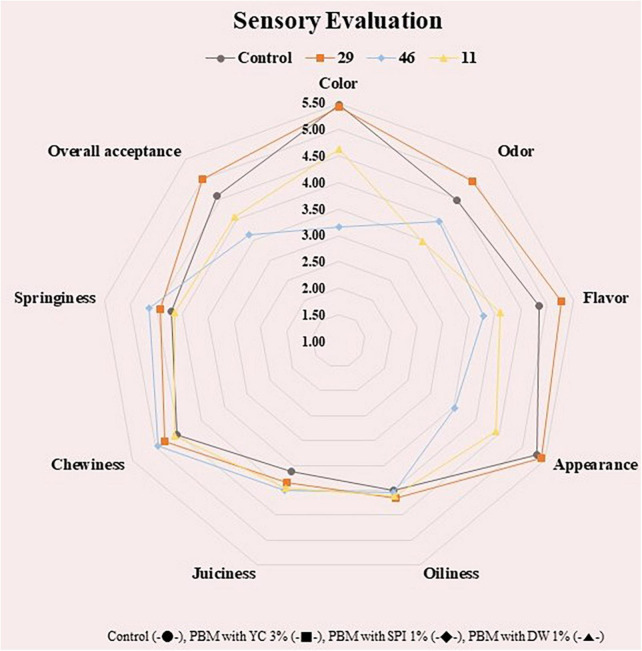
Sensory evaluation of PBM incorporated with novel microalgae ingredients. Control (-•-), PBM with YC 3% (-■-), PBM with SPI 1% (-◆-), PBM with DW 1% (-▲-).

One of the most important aspects of using microalgal plants in food systems is related to the palatability of microalgae proteins in terms of sensory input induced by aroma, color, taste, and textural mouthfeel (62). The sensory evaluation of PBM incorporated with various concentrations of microalgal protein has been shown in Figure 3.

In the sensory evaluation, there was no significant difference among the treatment groups (*p* > 0.05), nevertheless, SPI 1 showed the lowest value in color and appearance (*p* < 0.05) compared to the control. This agrees with the chromaticity outcome in Table 2 with redness values tending to be lowest in the SPI group. There was no significant difference in flavor, taste, and overall preference among the treatment groups (*p* > 0.05), which suggests the possibility of using SPI, DW, and YC as PBM additives. Previous literature indicated a similar distribution of sensory characteristics in beef patties replaced with pulses and algal proteins which did not affect the sensory profile (22). The outcome is revealing that the integration of soy and algal proteins could not make a substantial effect on the taste profile of beef patties. Whereas YC 3 was significantly brighter in saturation, but not significant in red and yellow, like the control, therefore, it is considered the best ingredient in the current formulation of PBM.

Furthermore, other studies have reported that the addition of a high concentration of microalgae and algal proteins might result in negative effects on the color and flavor of the final product, which depends on algae species and end product, decreasing consumers’ acceptance (63). For this reason, a concentration of a maximum of 5% (w/w) of algal biomass has been generally utilized and incorporated in algal-food products (32). To minimize the off-flavors in algal food products, the appropriate selection of algal strain, screening, and optimization of culture conditions, phytochemical studies, and characterization of odorant compounds are some key steps toward the successful development of the algal-food product (64–66). Similarly, SPI 1 and YC 3 showed excellent chewiness and gumminess, and YC 3 showed better effects than the control in the sensory evaluation. In addition, in the color measurement, the redness of meat was similar when comparing the DW and YC groups with commercial beef. The overall acceptance in the sensory evaluation were similar for all treatment groups, so suggesting that SPI, DW, and YC may be used to study the taste, color, and texture of PBM.

### Micronutrients and trace elements

A major challenge in developing PBM is the assurance of the nutritional profile of innovative products. Meat encompasses broader levels of proteins, as well as essential micronutrients, such as zinc, iron, and vitamin B. Besides, these micronutrients are frequently present in a highly bioavailable form within animal products (67). Therefore, it is essential to prepare PBM that are supplemented with bioavailable forms of these micronutrients. Consequently, in the current study, novel formulae were designed with the incorporation of microalgae protein in PBM patties (Table 5).

**TABLE 5:** Micronutrient of PBM incorporated with microalgae ingredients.

	Treatment									
	Control^1^	SPI 0.5^2^	SPI 0.7^3^	SPI 1^4^	DW 0.5^5^	DW 0.7^6^	DW 1^7^	YC 1^8^	YC 2^9^	YC 3^10^
Na (%)	0.38 ± 0.03^bc^	0.38 ± 0.06^bc^	0.41 ± 0.03^bc^	0.42 ± 0.03^c^	0.35 ± 0.03^abc^	0.37 ± 0.03^bc^	0.3 ± 0.02^a^	0.34 ± 0.01^ab^	0.35 ± 0.01^ab^	0.41 ± 0.03^cd^
K (%)	0.18 ± 0.03^a^	0.2 ± 0.01^a^	0.21 ± 0.03^a^	0.2 ± 0.02^a^	0.22 ± 0.01^a^	0.24 ± 0.02^a^	0.3 ± 0.025^b^	0.18 ± 0.03^a^	0.19 ± 0.01^a^	0.24 ± 0.06^a^
Zn (mg/kg)	27.92 ± 0.06^ab^	41.26 ± 1.06^bc^	45.31 ± 2.86^cd^	49.72 ± 5.46^cd^	16.99 ± 5.95^a^	25.33 ± 6.92^ab^	46.67 ± 8.3^cd^	39.34 ± 7.76^bv^	51.46 ± 2.33^cd^	58.15 ± 9.46^d^
Mn (mg/kg)	10.65 ± 0.04^a^	10.84 ± 0.5^a^	10.45 ± 1.07^a^	15.36 ± 2.64^a^	32.65 ± 0.65^b^	20.35 ± 7.28^a^	18.65 ± 12.57^a^	11.02 ± 2.28^a^	15.94 ± 9.46^a^	42.27 ± 7.76^b^
Fe (mg/kg)	36.11 ± 0.01^b^	22.21 ± 1.43^a^	23.02 ± 3.73^a^	24.87 ± 4.14^ab^	23.29 ± 10.35^a^	28.09 ± 4.61^cd^	50.45 ± 2.46^c^	23.4 ± 1.8^a^	35.33 ± 6.73^b^	64.48 ± 5.76^d^
Mg (mg/kg)	386.05 ± 3.65^ab^	450.29 ± 3.73^abc^	456.16 ± 14.21^abc^	469.92 ± 4.14^bc^	372.72 ± 8.88^a^	534.07 ± 46.03^c^	644.21 ± 31.14^d^	476.72 ± 10.4.92^bc^	480.4 ± 25.77^bc^	761.15 ± 0.85^e^
Cd	ND	ND	ND	ND	ND	ND	ND	ND	ND	ND
Hg (ppm)	0.01 ↓	0.01 ↓	0.01 ↓	ND	0.01 ↓	0.01 ↓	0.01 ↓	ND	ND	ND
As	ND	ND	ND	ND	ND	ND	ND	ND	ND	ND

ND = not detected. The results shown as mean ± standard deviation (n = 2). ^1^Control: PBM without treatment, ^2^SPI 0.5: 0.5% of Spirulina in PBM, ^3^SPI 0.7: 0.7% of Spirulina in PBM, ^4^SPI 1: 1.0% of Spirulina in PBM, ^5^DW 0.5: 0.5% of Duck Weed in PBM, ^6^DW 0.7: 0.7% of Duck Weed in PBM, ^7^DW 1: 1.0% of Duck Weed in PBM, ^8^YC 1: 1.0% of Yellow Chlorella in PBM, ^9^YC 2: 2.0% of Yellow Chlorella in PBM, ^10^YC 3: 3.0% of Yellow Chlorella in PBM. The results shown as mean standard deviation (n = 2). ^a,b,c,d,e,f^Means with the different letter are significantly different (p < 0.05).

The highest level of Na was detected in SPI 1 and the lowest in DWI, while K and Mn is displaying a higher trend in DW 1 and YC 3, respectively. Similarly, Zn had the highest values in YC 3 and the lowest in DW 0.5. Likewise, Fe and Mg were significantly higher in YC 3 and SPI 0.5 treated samples, and the lowest concentration for both elements was detected in SPI 0.5 and DW 0.5, respectively. Moreover, Cd, Hg, and As were either undetected or detected below 0.01. The current results demonstrated that micronutrients tended to be increased as concentration levels increased in treated samples. Micronutrients analyzed for Na, K, Zn, Mn, Fe, Mg, Cd, Hg, As, Na, and K did not differ significantly. However, consistent with the current results (68) reported that SPI was rich in Zn, and our results also showed high values for SPI 1 and YC 3. Previously, the technological and nutritional quality of bread wheat pasta supplemented with SPI has been investigated, and the outcomes of the study demonstrated that consuming SPI-based food had promising results for protein, minerals, and phenolic compounds (69). The literature regarding the incorporation of microalgae into PBM remains limited, particularly regarding micronutrients. However, some reports suggested that SPI, DW, and YC are rich in Mn and Mg (59, 70, 71).

### BCA and protein contents

**FIGURE 4:**
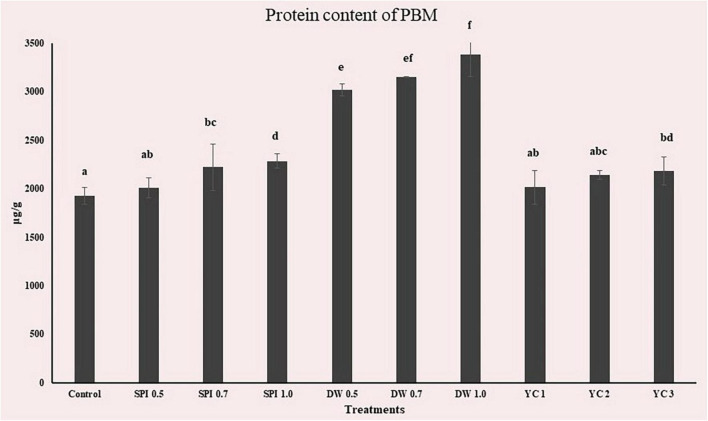
BCA analysis of PBM incorporated with novel microalgae ingredients. The results shown mean ± standard deviation (*n* = 4). ^a,b,c,d,e,f^Means with the different letters above a bar are significantly different (*p* < 0.05).

The protein concentration of the PBM incorporated with novel microalgae protein are presented in Figure 4. Standard curves were determined based on Bovine Serum Albumin (BSA) (*R*2 = 0.9988). The BCA analysis demonstrates the most significant change in the DW group of PBM. Conversely, SPI 0.5 (2004.633 μg/g), YC 1 (2013.98 μg/g), and YC 2 (2140.98 μg/g) did not change significantly (*p* > 0.05) compared to Control (1924.41 μg/g). As SPI, DW, and YC displayed a higher level of protein than the control hence these algal ingredients are more likely to be incorporated as functional and protein sources in future PBM products.

In supporting literature previously Suter (71) determined that SPI contains 63% protein and DW contains 29.05% while (59) reported that YC contains 55.76%. In contrast, the DW group with the lowest protein content depended on the concentration, followed by the SPI and YC groups. This is equivalent to the consumption of about 19% to 33% protein per 100 g of PBM. These results suggest that higher concentrations of DW led to increased protein content. Higher protein content in SPI and YC was reported in beef in patties replaced with alternative proteins (22). Hence, food products incorporated with microalgae have attracted the attention of investigators due to their extraordinary protein content which is reflected to be one of the tremendous protein sources of microalgal origin, with protein levels equivalent to that in conventional meat (13). Additionally, proteins and microalgae are the foundation of numerous valuable compounds with health assistance such as polyunsaturated fatty acids, carbohydrates, vitamins, and essential minerals, which can increase the nutritional value of food products upon incorporation (72).

### DPPH radical scavenging activity

**FIGURE 5:**
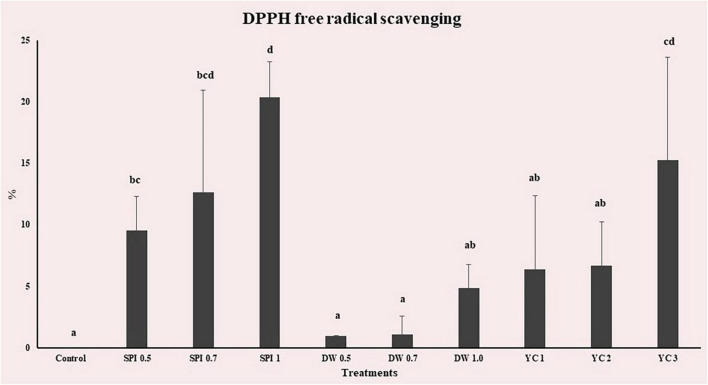
DPPH radical scavenging activity of PBM incorporated with novel microalgae. The results show mean ± standard deviation (*n* = 4). ^a,b,c,d,e,f^Means with the different letters above a bar are significantly different (*p* < 0.05).

The DPPH radical scavenging activity of PBM-containing treatment was measured based on standard curves with L-ascorbic acid (*R*2 = 0.9996). The results of measuring DPPH free radical scavenging ability on the antioxidant scale of PBM prepared by different amounts of treatment are shown in Figure 5. All groups were affected by treatment, and DPPH free radical scavenging ability increased as the higher concentration level was added. The control showed slight antioxidant activity. The highest free radical scavenging activity was detected in SPI 1 (20.36%) and the lowest free radical scavenging activity was observed in DW 0.5 (0.94%). Compared with the control, the DW group and YC 1, 2 showed no significant difference (*p* > 0.05). Antioxidant substances that delay or prevent the oxidation of substrate and their antioxidant activity eliminate reactive oxygen species (ROS) that are produced during metabolism. A higher concentration of free radicals in any specific formulation may lead to complications such as aging and inflammation (73). Therefore, antioxidant activity in food materials is important for the inhibition of higher levels of free radicals. The majority of previous studies have indicated that microalgae proteins including SPI, DW, and YC demonstrated antioxidant activity which is consistent with our results (61, 74–76). SPI 1 had the greatest ability to eliminate free radicals whereas the low antioxidant capacity of the DW group may be due to the loss of vitamin C due to hot water extraction (77).

Furthermore, SPI contains molecules such as phycocyanin, b-carotene, xanthophyll pigments, g-tocopherol, and phenolic compounds, which are responsible for the antioxidant activities of these microalgae (78, 79). The evidence of DPPH scavenging activity and antioxidant capacity of various microalgae (SPI and YC) incorporated into broccoli soup has been described (33). De Marco et al. (80) reported that pasta substituted with higher concentration SPI had higher antioxidant and phenolic content. There are many valuable antioxidants in YC, e.g., chlorophyll, carotenoids, astaxanthin, lutein, and phycobiliproteins (81). Furthermore, the protective effects of YC and its antioxidant activity are attributed to their content of phenolic compounds as there is a close positive relationship between the quantity of these compounds and their antioxidant activities due to their redox properties that play a vital role in capturing and scavenging free radicals, oxygen suppression and peroxide decomposition (82). Similarly, the DPPH radical scavenging of various types of YC were assessed in meat substitutes by Song et al. (83). Bioactive compounds such as carotenoids, phytosterols, and other pigments are naturally synthesized in duckweed with numerous antioxidants and anti-inflammatory properties that have been widely used in many foods and nutraceuticals (24, 84).

As PBM products are usually consumed after cooking by heating with added seasoning to improve taste. Song et al. (85) reported DPPH radical scavenging activity of meat substitutes increased by heating at various temperatures. The enhancement of DPPH radical scavenging activity was dependent on the heating temperature. The authors further demonstrate the increase in DPPH radical scavenging activity was also dependent on the temperature employed. As the temperature increased, the antioxidant activity of meat substitutes also increased. The possible reason could be, various reductones produced in the Maillard reaction process may contribute to the increased DPPH radical scavenging activity of meat substitutes by heating. Due to limited available information further argumentation about heating and DPPH radical scavenging activity of meat substitutes is not possible.

## Conclusion

Novel microalgae ingredients including SPI, DW, and YC, improved the physiochemical properties of PBM, and accordingly desirable results were obtained from the sensory evaluation. In contrast, the nutritional composition of PBM mixed with SPI, DW, and YC did not differ from each other. In addition, these ingredients showed better antioxidant activities than the control group. Owing to its antioxidant activity, PBM is expected to be a potential health-beneficial product. Moreover, micronutrient analysis indicated that PBM is heavy metal-free. Therefore the current results demonstrate that microalgae proteins could be useful candidates for the manufacture of novel alternative products. Further research is necessary to optimize the health functionality, texture, and palatability of PBM with these new ingredients.

## Data availability statement

The original contributions presented in this study are included in the article/supplementary material, further inquiries can be directed to the corresponding author.

## Author contributions

AB and JP: basic methodology, accomplish experiment, and writing the original draft. KB: visualization and supervision. BK: conceptualization, visualization, and data curation. SS: basic resources, methodology, and conceptualization. AR: revise and edit the manuscript. SP: basic methodology, funding acquisition, project administration, and writing—review and editing. All authors contributed to the article and approved the submitted version.
